# Supplementary figures and images for: Comparison of Transcriptional Signatures of Three Staphylococcal Superantigenic Toxins in Human Melanocytes
Source: Biomedicines. 2022 Jun 14;10(6):1402. doi: 10.3390/biomedicines10061402 (PMC9219963; doi:10.3390/biomedicines10061402)

# Figure S1

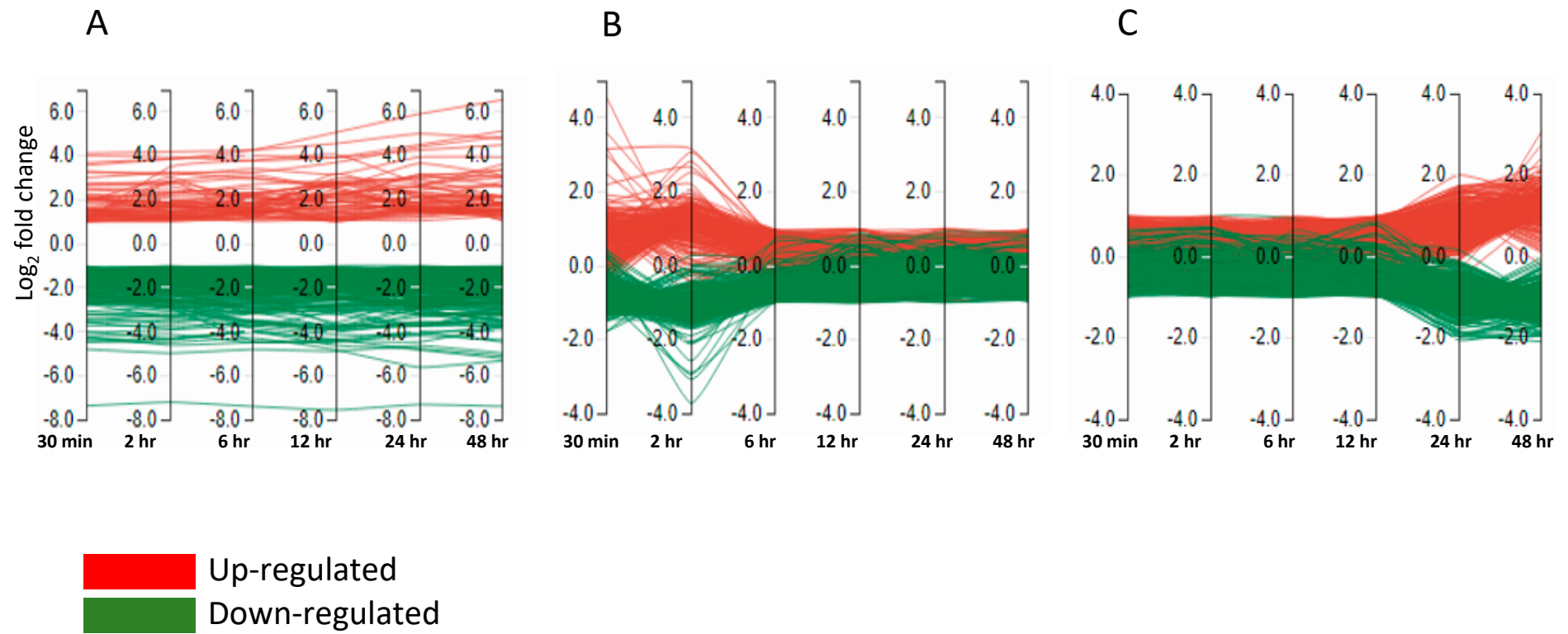

# Figure S2

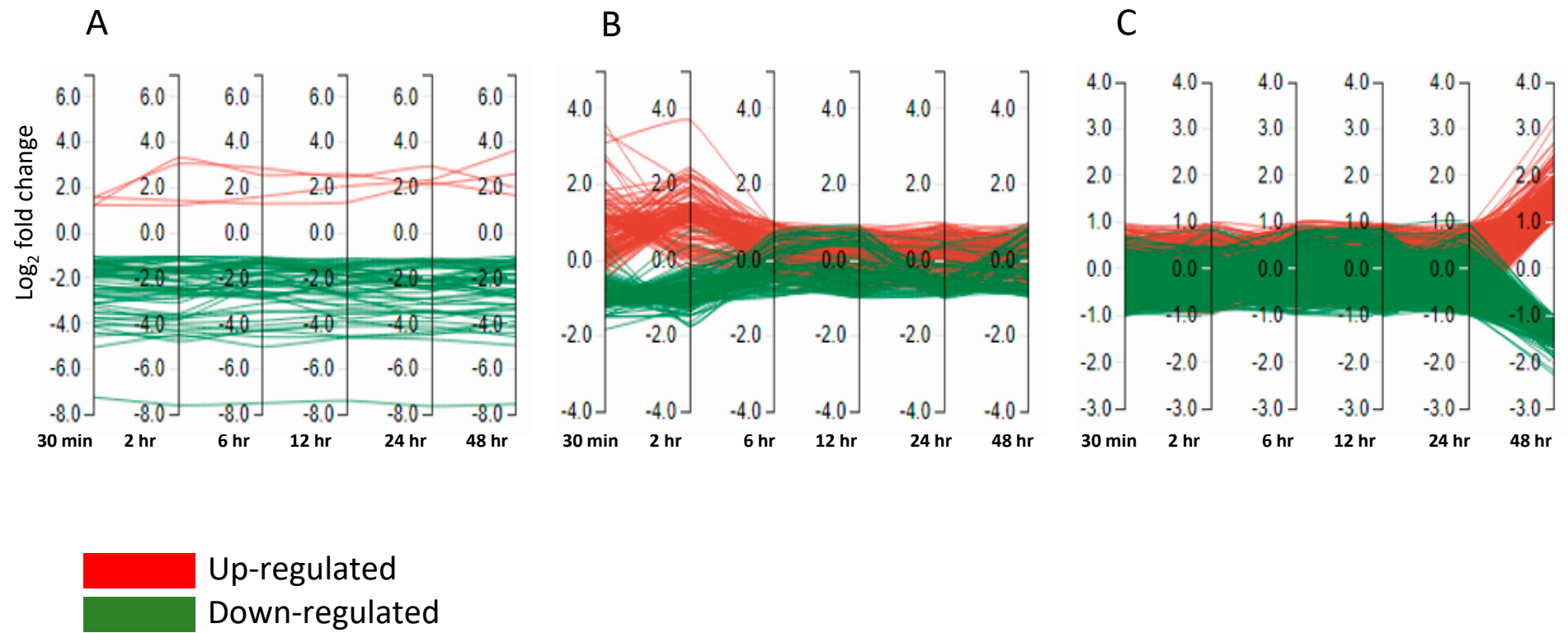

# Figure S3

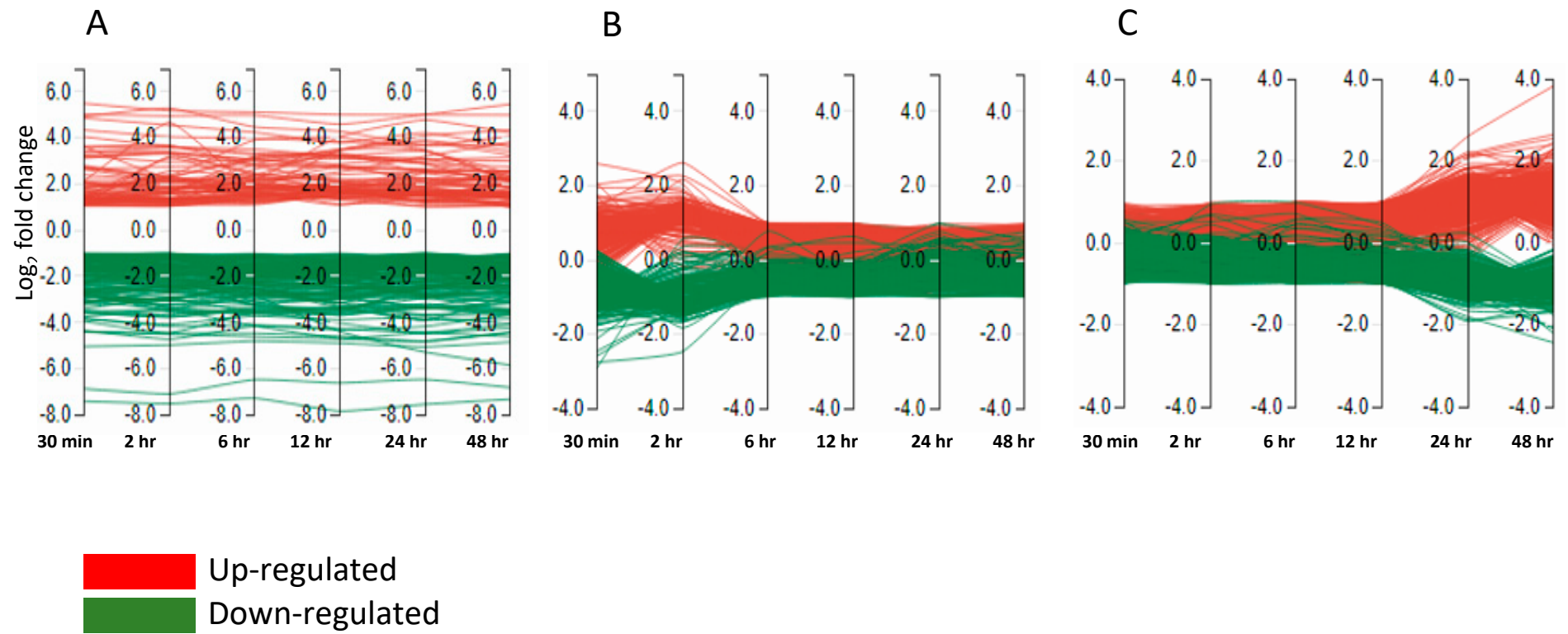

# Figure S4

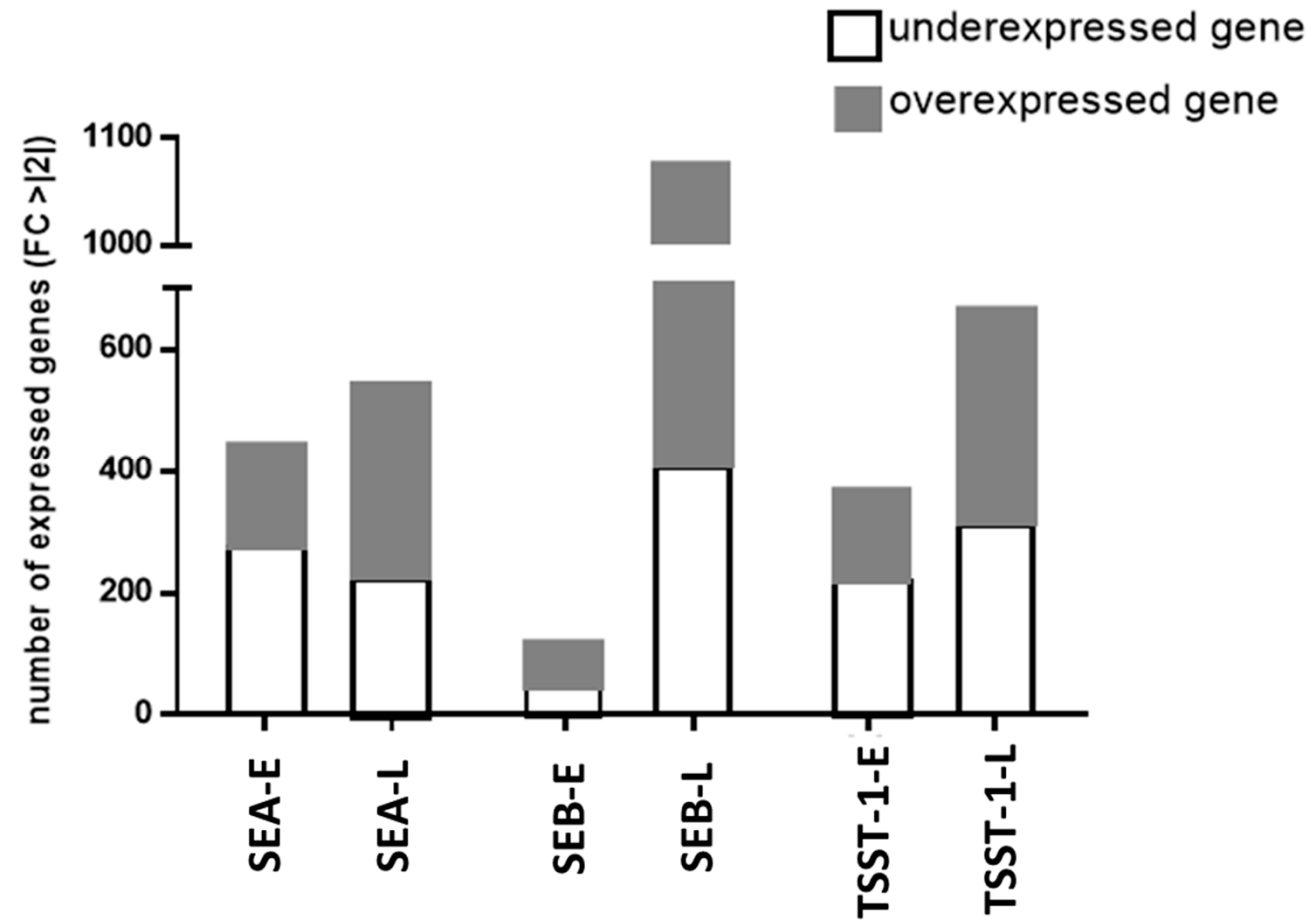

EARLY

# Figure S5A

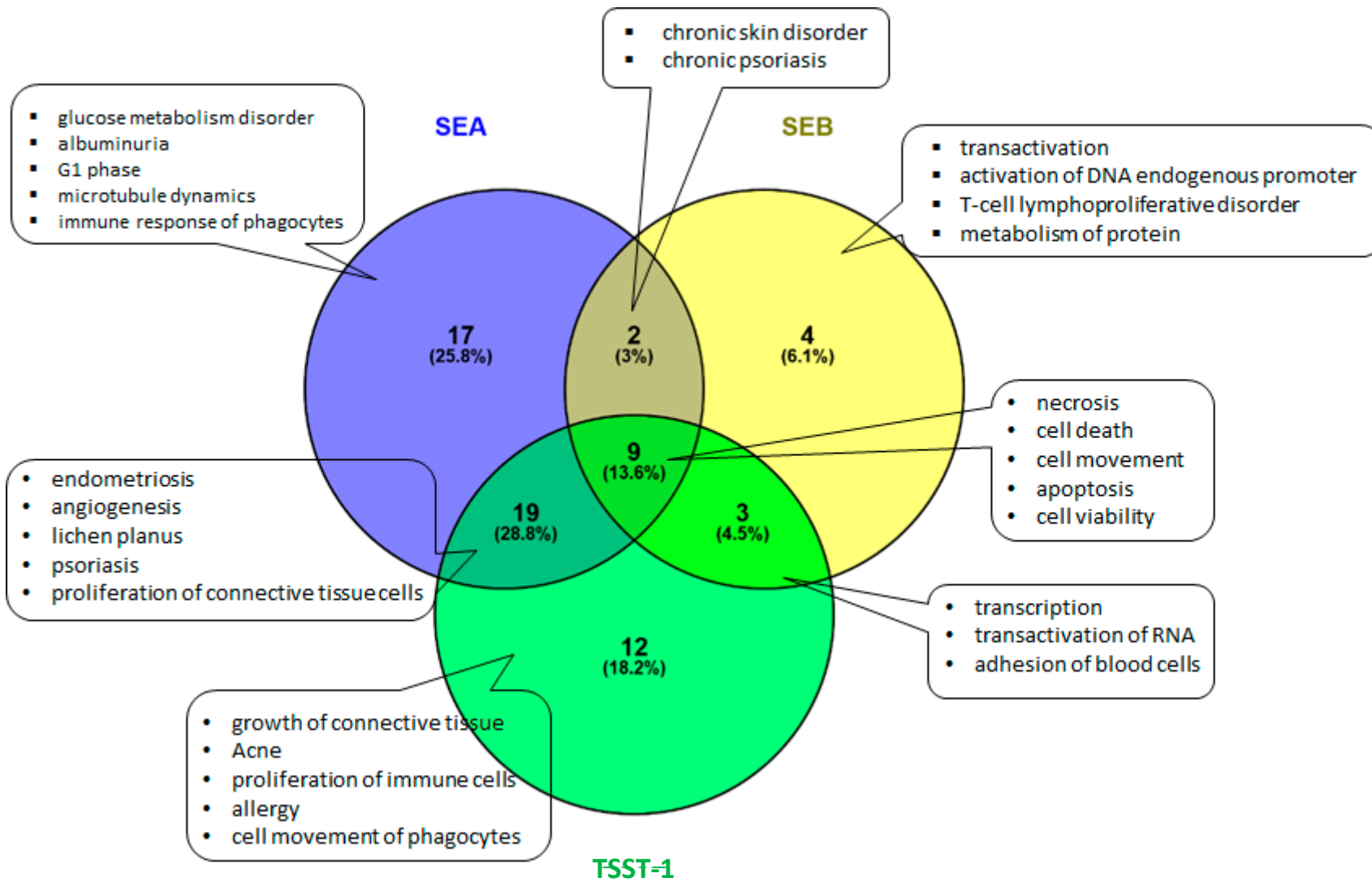

# Figure S5B

LATE

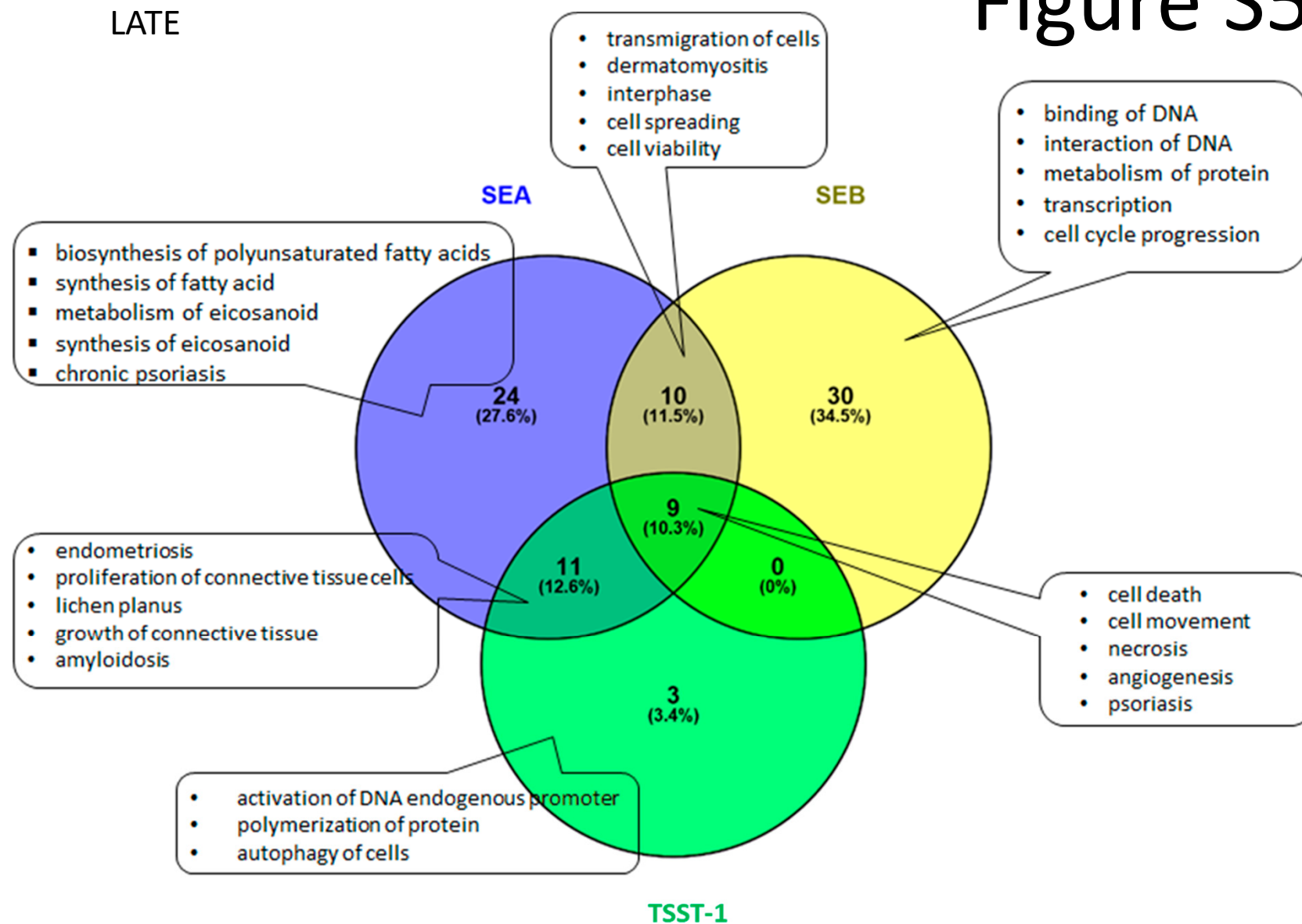

Figure S6

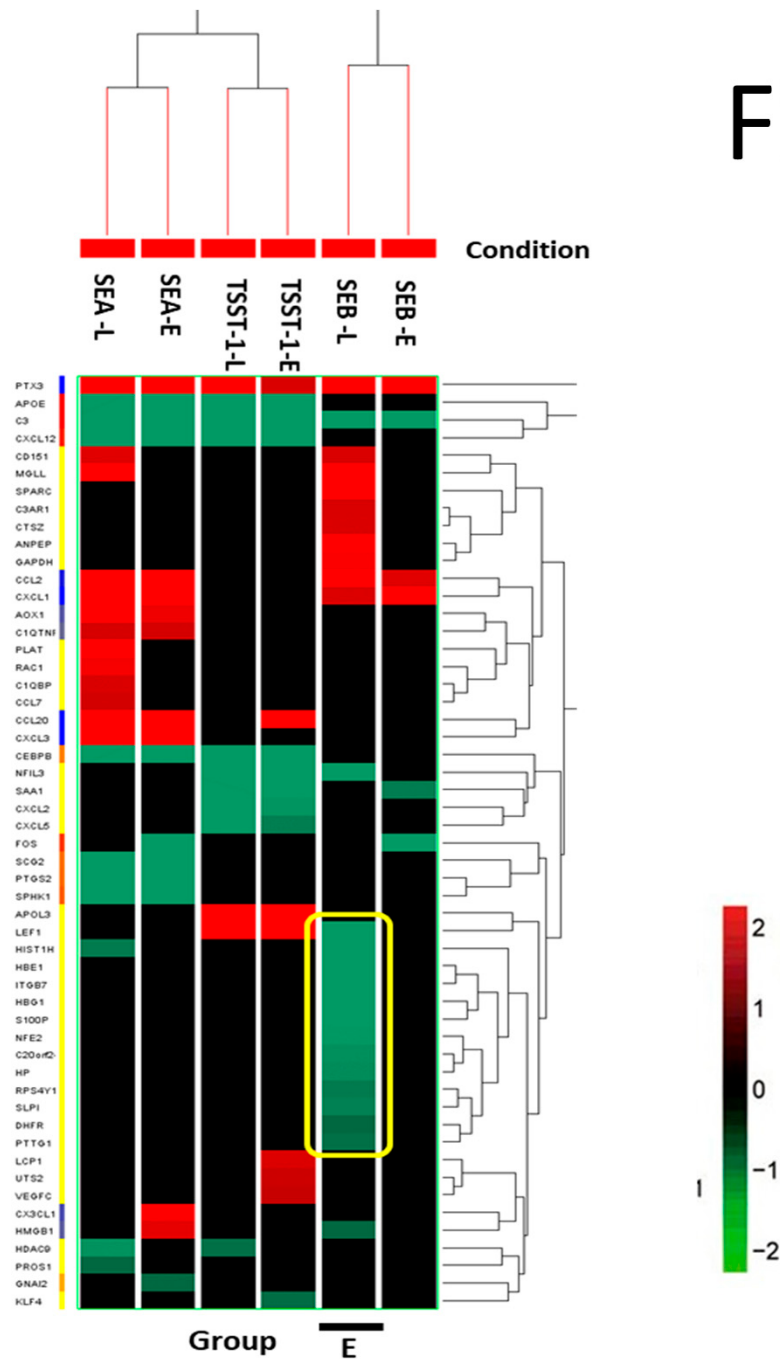

Figure S7

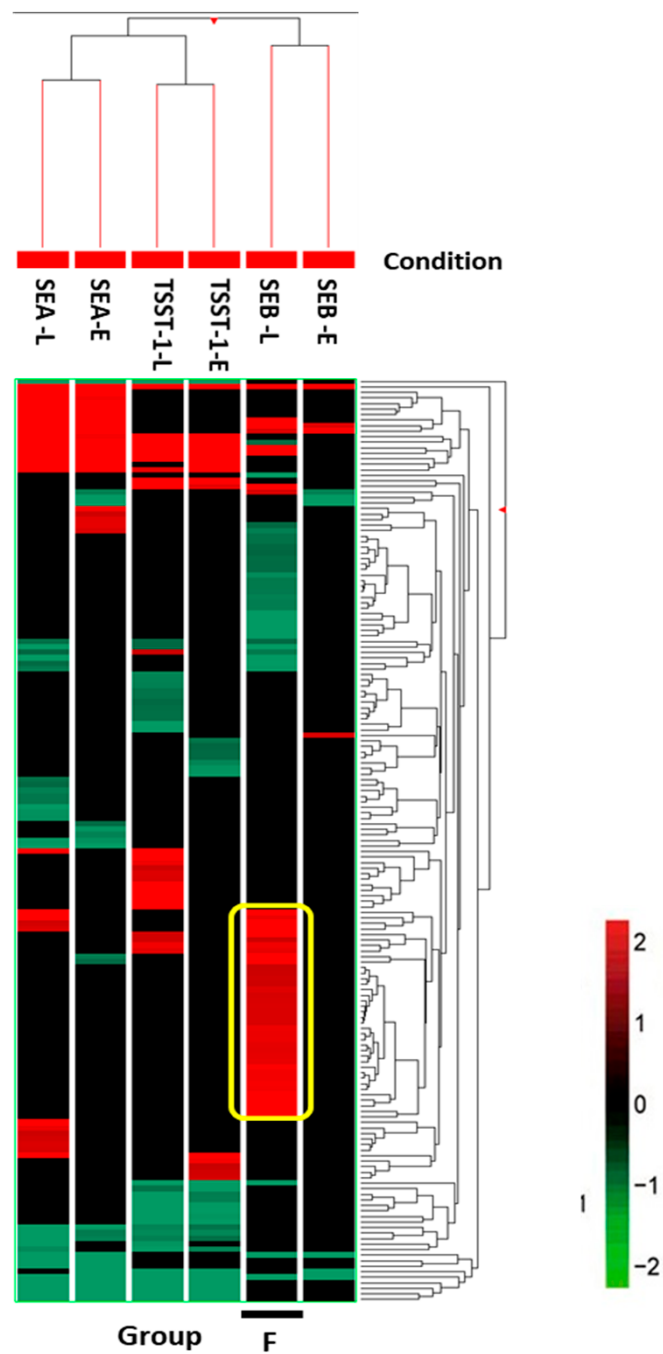

# Figure S8

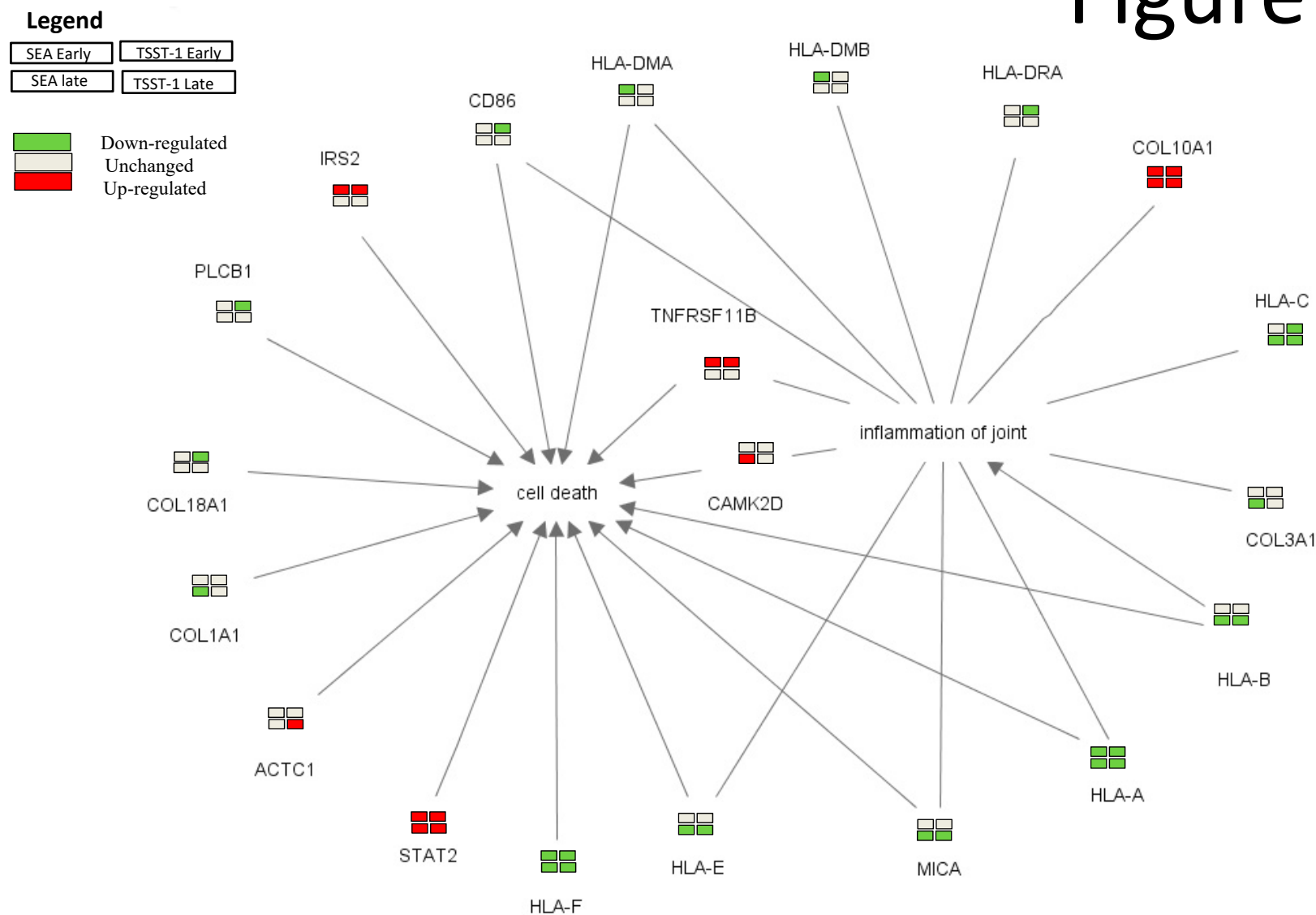

Supplement: Supplementary file 1 [file biomedicines-10-01402-s001.zip › Supplementary Figures.pdf]
